# Supplementary material for: A potent broadly neutralizing human RSV antibody targets conserved site IV of the fusion glycoprotein
Source: Nat Commun. 2019 Sep 12;10:4153. doi: 10.1038/s41467-019-12137-1 (PMC6742648; doi:10.1038/s41467-019-12137-1)
Supplement: Supplementary file 1 — Supplementary Information [file 41467_2019_12137_MOESM1_ESM.pdf]

Supplementary Files

Tang, et al.

A potent broadly neutralizing human RSV antibody targets conserved site IV of the fusion glycoprotein

Supplementary Figure 1: Alignment of RB1 variable regions with germline sequences

```
                        |         |          |         |         |        |         |
        RB1_VH  EVQLVESGGGLVRPGRSLRLSCTVSGFSFDDSAMSWVRQAPGKGLEWISFIKSKTYGGTKEYAASVKGRF
   IGHV3-49*04  ............Q..........A...T.G.Y...............VG..R..A....T..........

                        |         |          |         |         |
        RB1_VH  TISRDDSKNIAYLQMNSLKTEDTAVYYCTRGAPYGGNSDYYYGLDVWGQGTTVTVSS
   IGHV3-49*04  ........S.....................
     DH4-23*01                                   .....
       JH6*02                                          ....M.............

                        |         |          |         |         |        |         |
        RB1_VL  DIQMTQSPSSLSASVGDRVTITCRTSQDVRGALAWYQQKPGKAPKLLIFDASSLETGVPSRFSGSGSGTV
  IGKV1D-13*01  A..L....................A..GISS..................Y......S.............D

                 |         |          |         |
        RB1_VH  FTLTISSLQPEDFAAYYCQQFLDFPFTFGQGTRL
  IGKV1D-13*01  ..............T......NNY.
       JK5*01                     ....X.. ........
```

Figure S1: Alignment of RB1 variable regions with germline sequences

The amino acid sequence of the RB1 antibody was aligned to the human antibody germline sequence using the IgBlast (https://ftp.ncbi.nih.gov/blast/executables/igblast/release/LATEST) program and the antibody complementarity-determining region (CDR) of the heavy chain (shown as RB1_VH) and the light chain (shown as RB1_VK) were determined according to Kabat numbering system and sequentially highlighted in colors.

Supplementary Figure 2: Surface plasmon resonance (SPR) analysis of RB1 binding to pre-fusion and post-fusion RSV F proteins

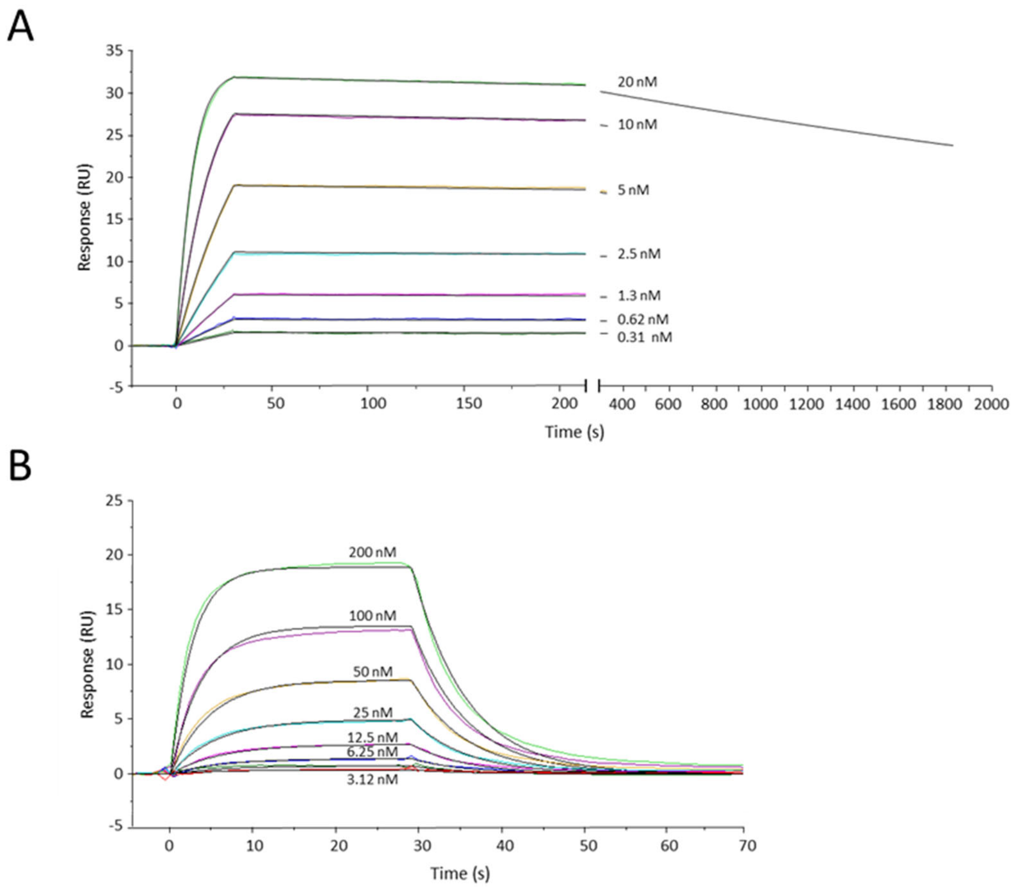

Figure S2: Surface plasmon resonance (SPR) analysis of RB1 binding to pre-fusion and post-fusion RSV F proteins.

A) Pre-fusion (DS-Cav-1) protein was captured by D25 antibody previously loaded on a Biacore Series S Sensor Chip Protein A chip. Two-fold serial dilutions of monovalent RB1 monovalentFab were flowed for 30 sec followed by a dissociation period of 300 sec (0.31 nM to 10 nM) or 1800 sec (20 nM). Kinetic and equilibrium constants were determined by fitting the data sets to a 1:1 Langmuir binding model. B) RSV post fusion protein was captured by Synagis antibody previously loaded on a Biacore Series S Sensor Chip Protein A chip. Two-fold serial dilutions of monovalent RB1 Fab (200 nM to 3.12 nM) were flowed for 30 sec followed by a dissociation period of 40 sec. Kinetic and equilibrium constants were determined by fitting the data sets to a 1:1 Langmuir binding model. RU = Resonance Units.

Supplementary Figure 3: Identification of critical residues for monoclonal antibody RB1 binding using shotgun mutagenesis epitope mapping

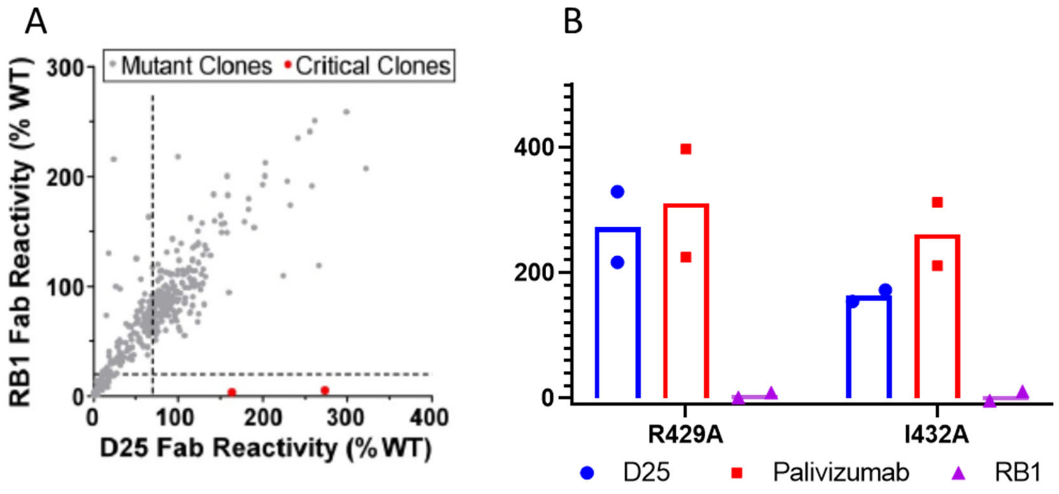

Figure S3: **Identification of critical residues for monoclonal antibody RB1 binding using shotgun mutagenesis epitope mapping.**

A shotgun mutagenesis alanine scanning library was constructed for the RSV F protein. The library contains 368 individual mutations at residues identified as surface exposed on the pre-fusion and post-fusion forms of RSV F proteins. Each well of the mutation array plate contained one mutant with a defined substitution. (A) Human HEK293T cells expressing the RSV F mutation library were tested for immunoreactivity with RB1, measured by flow cytometry. Clones with reactivity of <15% relative to that of wildtype RSV F (horizontal line) yet >70% reactivity for a control monoclonal antibody were identified to be critical for RB1 binding (red dots). (B) Mutation of two individual residues reduced RB1 binding but not the binding of D25 and palivizumab. The bar represents the mean of each of the two replicates that were performed and each of the replicate data points are also shown.

Supplementary Figure 4: Stereo view of electron density

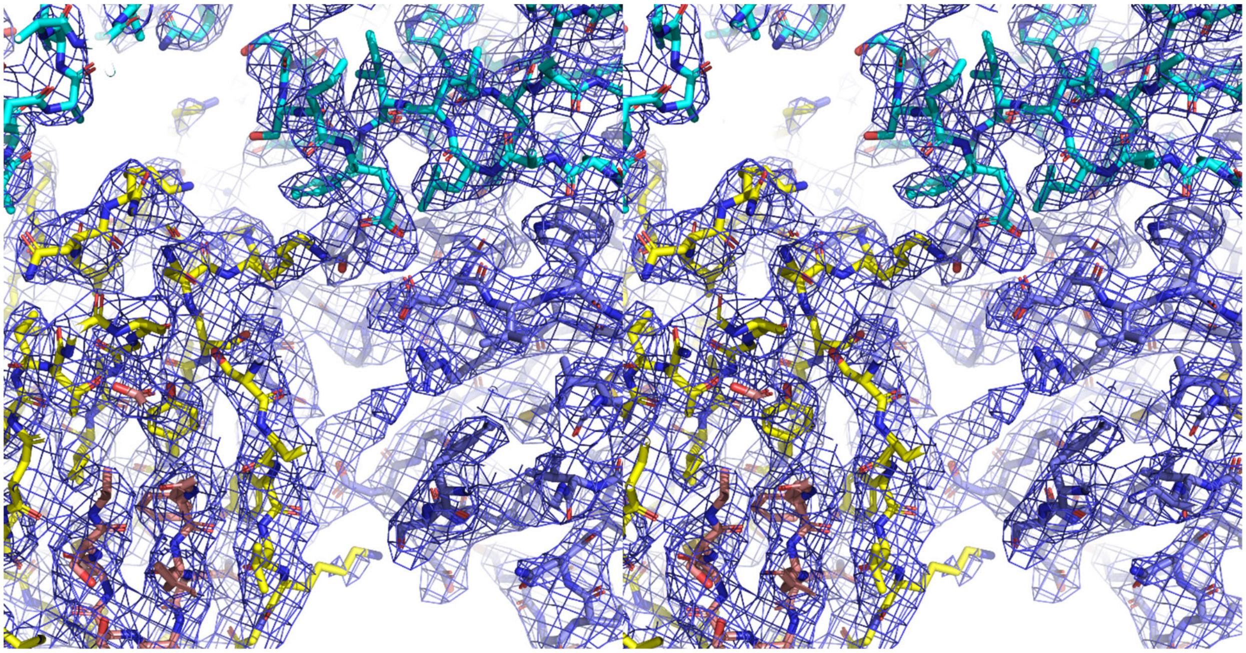

Figure S4: Stereo view of electron density of the RB1 and Pre-fusion F binding

Stereo image of $2F_o$-F_c electron density map contoured at 1σ around the interface between heavy chain (blue), light chain (cyan) and RSV-F (yellow).

Supplementary Figure 5: **RSV Fusion Protein Sequence profile of each amino acid position**

Supplemental Figure 5A RSV F Protein Sequence Profile: amino acids 1 to 300

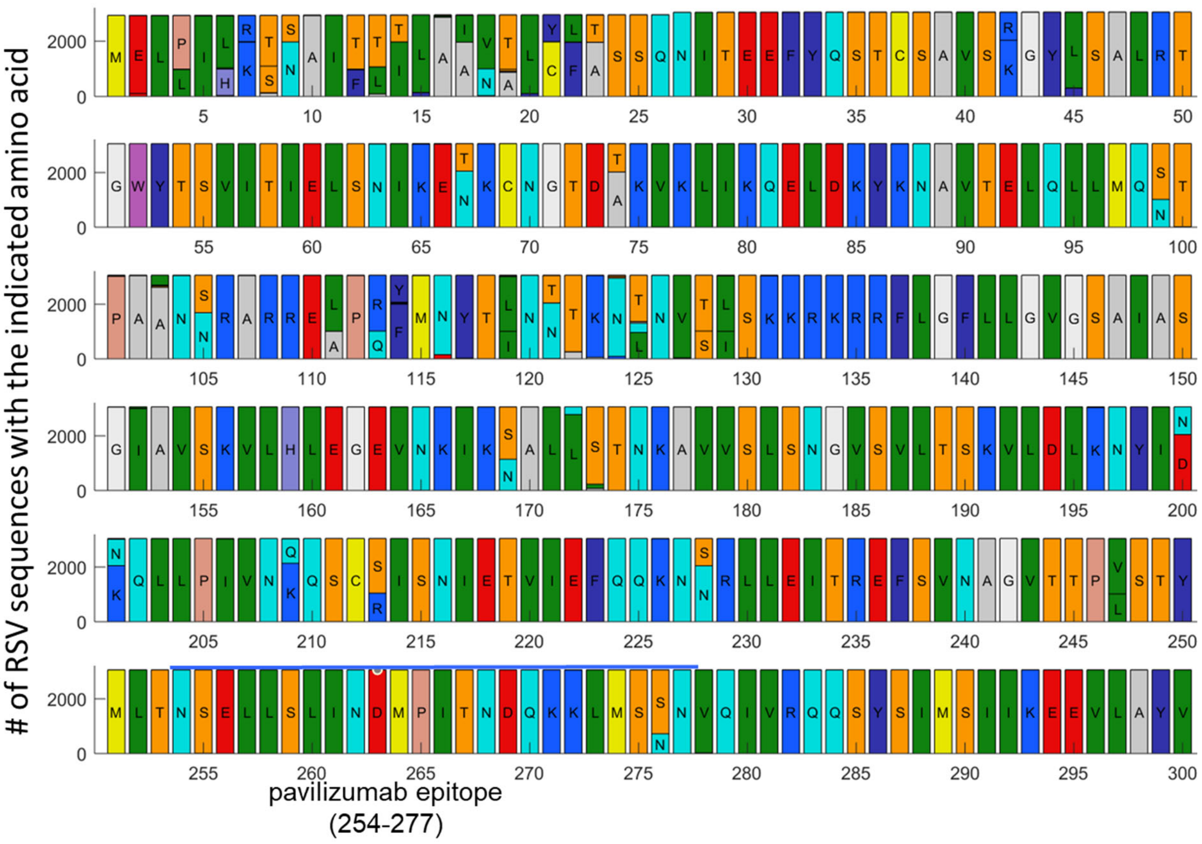

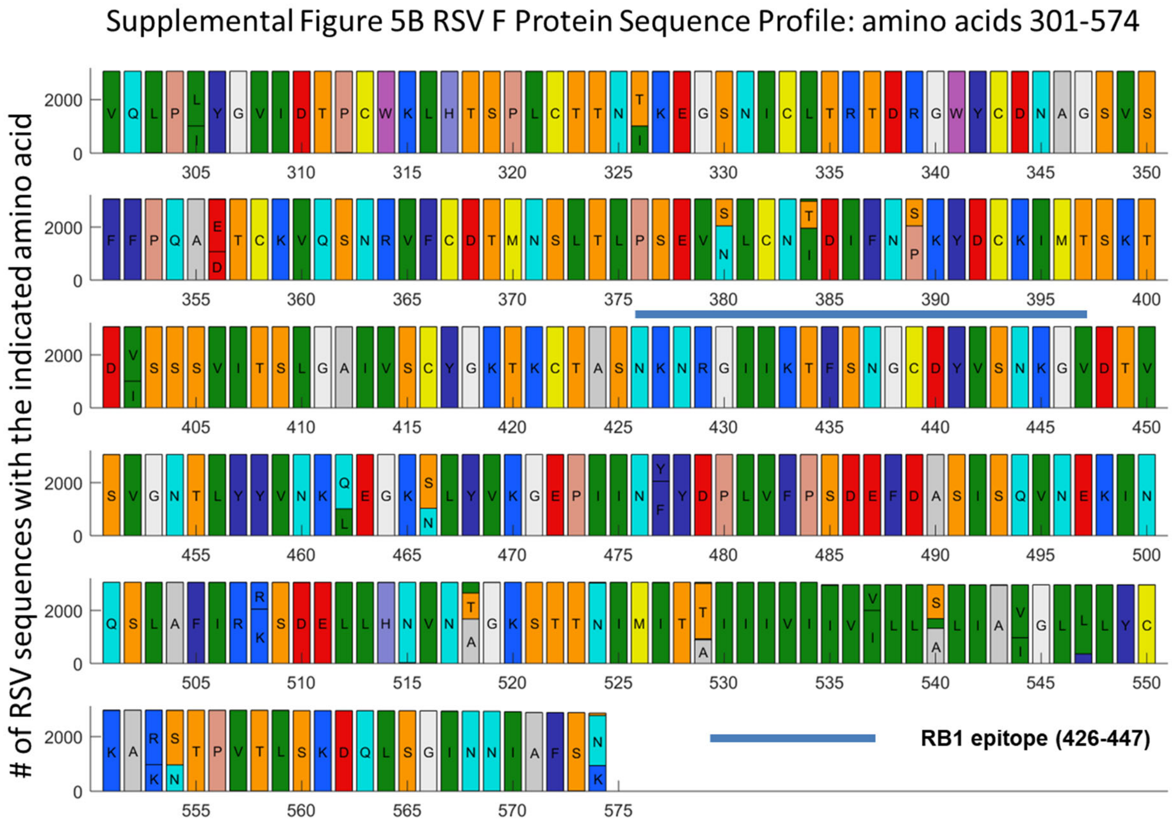

Figure S5: **RSV Protein Sequence profile of each amino acid position of the Fusion protein**

A sequence diversity evaluation was performed for each amino acid of the RSV Fusion protein. 3,058 complete RSV F glycoprotein sequences were obtained from GenBank for analyses (https://www.ncbi.nlm.nih.gov/genbank/); accessed April 2019). The sequences were aligned, and the percent identity was determined. A logo plot depicts the amino acid identities at each position for amino acids 1 through 300 (A) and 301 through 574 (B) on the X axis and the Y axis indicates the number of RSV sequences. The amino acids in the logo plot are colored according to their physicochemical properties according to the RasMol color scheme (Rasmol v2.6) where the height of each part of the bar indicates the number of sequences with the indicated amino acid(s) at the position.

Supplementary Figure 6: ELISA binding of RB1 to MARMS

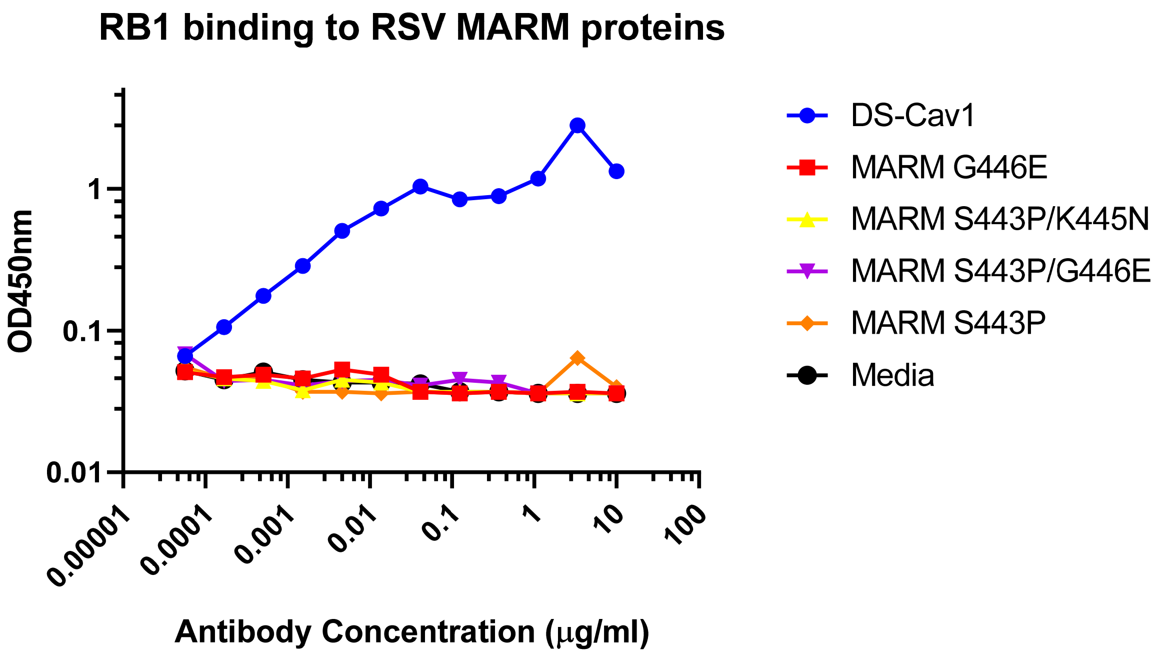

Figure S6: **Enzyme linked Immunoassay (ELISA) binding to RSV F proteins with mutations found in the MARMS generation**

RSV F protein of pre-fusion F (DS-Cav1) or mutants based on the MARMS sequences were used to determine the ability of these proteins to bind with RB1. Sequences of the MARMS are based on a DS-Cav1 background with either one or two mutations, as listed in the legend. The protein was detected with a titration of RB1 in a single replicate.

Supplementary Figure 7: Viral growth curves of RB1 MARMS

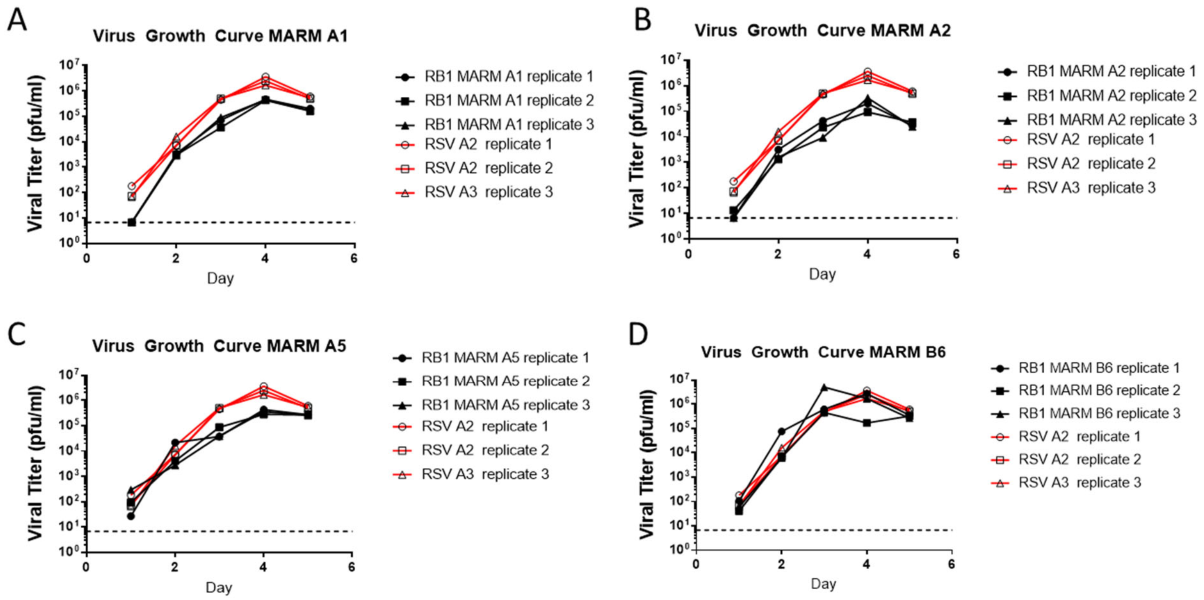

Figure S7: Viral growth curves of RB1 MARMS

Four monoclonal antibody-resistant mutant (MARM) RSV strain A viruses were identified for RB1 after extensive selective in vitro pressure with an RSV A Long laboratory strain. Each MARM virus was assessed for viral growth characteristics on HEp-2 cells. Culture supernatants were harvested on days 1, 2, 3, 4, and 5 after infection and assessed for viral titer (pfu/mL) using a plaque assay. Shown in each panel are the viral growth characteristics for MARM RB1-A1 (A), RB1-A2 (B), RB1-A5 (C), and RB1-B6 (D). Each assay was performed in triplicate. The dotted lines equal assay limit of detection.

Supplementary Table 1: Primer sequences for human B cell cloning

RT-PCR primers			
		Primer name	Primer sequence
Heavy chain	5' primer	5' LH1/7 For	5' ACAGGTGCCCACTCCCAGGTGCAG
		5'LH2 For	5' CTGACCATCCCTTCATGGGTCTTGTCC
		5' LH3 For	5' AAGGTGTCCAGTGTGARGTGCAGCTG
		5' LH4/6 For	5' AGATGGGTCCTGTCCCAGGTGCAG
		5' LH5 For	5' CAAGGAGTCTGTTCCGAGGTGCAGC
	3' primer	3' IgG JH Rev	5' GTGGAGGCTGARGAGACRGTGACC
Kappa chain	5' primer	5'LK1/2 For	5' ATGAGGGTCCCYGCTCAGCTCCTG
		5' LK3 For	5' CTCTTCCTCCTGCTACTCTGGCTC
		5' LK4 For	5' CAGGTCTTCATTTCTCTGTTGCTCTGG
		5' LK5 For	5' GTTCACCTCCTCAGCTTCCTCCTC
		5'LK6 For	5' CTCTGGGTTCCAGCCTCCAGG
	3' primer	3' Kappa Rev	5' ACACTCTCCCCTGTTGAAGCTCTTTGTG
Lambda chain	5' primer	5' Lλ1 For	5' GGTCCTGGGCCCAGTCTGTGCTG
		5' Lλ2 For	5' GGTCCTGGGCYCAGTCTGCCCTG
		5' Lλ3 For	5' GCTCTGWGGCCTCCTATGAGCTG
		5' Lλ4,5,9 For	5' GGTCTCTCTCSCAGCYTGTGCTG
		5' Lλ6 For	5' GTTCTTGGGCCAATTTTATGCTGACTC
		5' Lλ7 For	5' GGTCCAATTCYCAGGCTGTGGTG
		5' Lλ8 For	5' GAGTGGATTCTCAGACTGTGGTG
		5' Lλ10 For	5' GTGTCAGTGGTCCAGGCAGGGCTG
	3' primer	3' Lambda Rev	5' GCATTCTGYAKGGGCMAYTGTC
Nested-PCR primers			
		Primer name	Primer sequence
Heavy chain	5' primer	5'VH1,7 For	5'CTGCCCAACCAGCCATGGCCCAGGTGCAGCTGGTGCAGTCTGG
		5'VH2 For	5'CTGCCCAACCAGCCATGGCCCAGATCACCTTGAAGGAGTCTGG
		5'VH3,5 For	5'CTGCCCAACCAGCCATGGCCGAGGTGCAGCTGGTGSAGTCTGG
		5'VH4 For	5'CTGCCCAACCAGCCATGGCCCAGGTGCAGCTGCAGSAGTCGGG
		5'VH6 For	5'CTGCCCAACCAGCCATGGCCCAGGTACAGCTGCAGCAGTCAGG
	3' primer	3' JH Nhe1 Rev	5' ACGCGCCGCACCGGTGCTAGCTGARGAGACRGTGACC
Kappa chain	5' primer	5' VK1a, b For	5'AATGGCCCAGGCGGCCGACATCCAGWTGACCCAG
		5' VK1c For	5'AATGGCCCAGGCGGCCGCCATCCGGTTGACCCAG
		5' VK2 For	5'AATGGCCCAGGCGGCCGATATTGTGATGACYCAG
		5' VK3a, b For	5'AATGGCCCAGGCGGCCGAAATTGTGTTGACRCAG
		5' VK3c For	5'AATGGCCCAGGCGGCCGAAATAGTGATGACGCAG
		5' VK4 For	5'AATGGCCCAGGCGGCCGACATCGTGATGACCCAG
		5' VK5 For	5'AATGGCCCAGGCGGCCGAAACGACACTCACGCAG
		5' VK6a For	5'AATGGCCCAGGCGGCCGAAATTGTGCTGACTCAG
		5' VK6b For	5'AATGGCCCAGGCGGCCGATGTTGTGATGACACAG
	3' primer	3' CK BsiW1 Rev	5'GATGGTGCAGCCACCGTACGTTTRATHTCC
Lambda chain	5' primer	5' VL1 For	5'AATGGCCCAGGCGGCCCAGTCTGTGCTGACKCAG
		5' VL2 For	5'AATGGCCCAGGCGGCCCAGTCTGCCCTGACTCAG
		5' VL3 For	5'AATGGCCCAGGCGGCCTCCTATGAGCTGACWCAG
		5' VL4,5,9 For	5'AATGGCCCAGGCGGCCCAGCYTGTGCTGACTCA
		5' VL6 For	5'AATGGCCCAGGCGGCCAATTTTATGCTGACTCAG
		5' VL7,8 For	5'AATGGCCCAGGCGGCCCAGRCTGTGGTGACTCAG
		5' VL10 For	5'AATGGCCCAGGCGGCCCAGGCAGGGCTGACTCAG
	3' primer	3' CL Xho1 Rev	5'CTCCTCACTCGAGGGTGGGAACAGAGTGACAG

Overlap-PCR primers		
	Primer name	Primer sequence
5' primer	5' Infusion Kappa/Lambda F	5' TCGCTACCGTGGCCCAGGCGGCC
3' primer	3' Infusion VH R2	5' GGCCTTTGGTGCTAGCTGARGAGACRGTG

Linker	
Linker name	Linker sequence
CKappa/VH linker	5'CGTACGGTGGCTGCACCATCTGTCTTCATCTTCCCGCCATCTGATGAGCAGTTGAAATCTGGAACTGCCTCTGTTGTGTGCCTG CTGAATAACTTCTATCCCAGAGAGGCCAAAGTACAGTGGAAGGTGGATAACGCCCTCCAATCGGGTAACTCCCAGGAGAGTGT CACAGAGCAGGACAGCAAGGACAGCACCTACAGCCTCAGCAGCACCCTGACGCTGAGCAAAGCAGACTACGAGAAACACAAA GTCTACGCCTGCGAAGTCACCCATCAGGGCCTGAGCTCGCCCGTCACAAAGAGCTTCAACAGGGGAGAGTGTTCTAGATGATA AGCATGCGTAGGAGAAAATAAAATGAAATACCTATTGCCTACGGCAGCCGCTGGATTGTTATTACTCGCTGCCCAACCAGCCAT GGC

CLambda/VH linker	5’CTGTTCCCACCCTCGAGTGAGGAGCTTCAAGCCAACAAGGCCACACTGGTGTGTCTCATAAGTGACTTCTACCCGGGAGCCG<br>TGACAGTGGCCTGGAAGGCAGATAGCAGCCCCGTCAAGGCGGGAGTGGAGACCACCACACCCTCCAAACAAAGCAACAACAA<br>GTACGCGGCCAGCAGCTATCTGAGCCTGACGCCTGAGCAGTGGAAGTCCCACAGAAGCTACAGCTGCCAGGTCACGCATGAA<br>GGGAGCACCGTGGAGAAGACAGTGGCCCCTACAGAATGTTCTAGATGATAAGCATGCGTAGGAGAAAATAAAATGAAATACC<br>TATTGCCTACGGCAGCCGCTGGATTGTTATTACTCGCTGCCCAACCAGCCATGGC

K = G / T, M = A / C, R = A / G, S = G / C, W = A / T, Y = C / T, H = A / C / T.

For= forward, Rev= reverse
