# Supplementary material for: A potent broadly neutralizing human RSV antibody targets conserved site IV of the fusion glycoprotein
Source: Nat Commun. 2019 Sep 12;10:4153. doi: 10.1038/s41467-019-12137-1 (PMC6742648; doi:10.1038/s41467-019-12137-1)
Supplement: Supplementary file 3 — Reporting Summary [file 41467_2019_12137_MOESM3_ESM.pdf]

nature research

Corresponding author(s): Kalpit A. Vora

Last updated by author(s): 07-Aug- 2019

Reporting Summary

Nature Research wishes to improve the reproducibility of the work that we publish. This form provides structure for consistency and transparency in reporting. For further information on Nature Research policies, see Authors & Referees and the Editorial Policy Checklist.

Statistics

For all statistical analyses, confirm that the following items are present in the figure legend, table legend, main text, or Methods section.

n/a	Confirmed	
☐	☒	The exact sample size (*n*) for each experimental group/condition, given as a discrete number and unit of measurement
☐	☒	A statement on whether measurements were taken from distinct samples or whether the same sample was measured repeatedly
☐	☒	The statistical test(s) used AND whether they are one- or two-sided<br>*Only common tests should be described solely by name; describe more complex techniques in the Methods section.*
☒	☐	A description of all covariates tested
☒	☐	A description of any assumptions or corrections, such as tests of normality and adjustment for multiple comparisons
☐	☒	A full description of the statistical parameters including central tendency (e.g. means) or other basic estimates (e.g. regression coefficient) AND variation (e.g. standard deviation) or associated estimates of uncertainty (e.g. confidence intervals)
☒	☐	For null hypothesis testing, the test statistic (e.g. *F*, *t*, *r*) with confidence intervals, effect sizes, degrees of freedom and *P* value noted<br>*Give P values as exact values whenever suitable.*
☒	☐	For Bayesian analysis, information on the choice of priors and Markov chain Monte Carlo settings
☒	☐	For hierarchical and complex designs, identification of the appropriate level for tests and full reporting of outcomes
☒	☐	Estimates of effect sizes (e.g. Cohen's *d*, Pearson's *r*), indicating how they were calculated

Our web collection on statistics for biologists contains articles on many of the points above.

Software and code

Policy information about availability of computer code

Data collection	Perkin Elmer Victor-Multi label counter (ELISA)<br>Li-Cor Aerius Automated Imaging system (neutralization assay)<br>Biacore 2000 , GE healthcare(SPR)<br>Canadian Light Source Synchrotron (Crystal structure)<br>Ensight plate reader , Perkin Elmer (HMPV neutralization assay)
Data analysis	Graphpad prism 6.0<br>BIA evaluation Software V4.1 (SPR)<br>Autoproc ,Global Phasing Limited (crystal structure)<br>Phaser as implemented in the phenix software package (crystal structure)<br>Buster, Global phasing limited (crystal structure)<br>Crystallographic object-oriented toolkit (Coot) , (crystal structure)<br>Seq2Logo (sequence analysis for Genbank sequences)<br>ClustalW (www.clustal.org) for Genebank sequence clustering<br>FigTree (http://tree.bio.ed.as/software/figtree/. - for dendrogram<br>Sequencher by gene codes corp.- for MARMS sequence analysis<br>Vector NTI , Invitrogen- MARMS sequence analysis<br>PyMOL, version 2.2 (for molecular data analysis)

For manuscripts utilizing custom algorithms or software that are central to the research but not yet described in published literature, software must be made available to editors/reviewers. We strongly encourage code deposition in a community repository (e.g. GitHub). See the Nature Research guidelines for submitting code & software for further information.

Data

Policy information about availability of data

All manuscripts must include a data availability statement. This statement should provide the following information, where applicable:

- Accession codes, unique identifiers, or web links for publicly available datasets
- A list of figures that have associated raw data
- A description of any restrictions on data availability

Merck & Co., Inc.'s data sharing policy, including restrictions, is available at http://engagezone.merck.com/ds_documentation.php. Requests for access to the study data can be submitted through the EngageZone site or via email to dataaccess@merck.com

Field-specific reporting

Please select the one below that is the best fit for your research. If you are not sure, read the appropriate sections before making your selection.

☒ Life sciences ☐ Behavioural & social sciences ☐ Ecological, evolutionary & environmental sciences

For a reference copy of the document with all sections, see nature.com/documents/nr-reporting-summary-flat.pdf

Life sciences study design

All studies must disclose on these points even when the disclosure is negative.

Sample size	Sample size: The hypothesized effect size and dose groups for each comparison was derived from historical or pilot study data. Internal statistics of data from various studies related (mAb) or unrelated (vaccine) were done. Similar studies, using similar powered groups were analyzed by ANOVA with Bonferroni correction (which simultaneously compares the efficacy at the each dose level between two compounds) in order to determine if differences exist. Moreover, previous documented studies use similar sized groups; Zhu, et al Sci Transl Med 2017; 9: DOI: 10.1128/AAC.00643-10; Boukhvalova et al. Bone Marrow Transplantation volume 51, pages 119–126 (2016); Boukhvalova et al Antiviral Chemistry and Chemotherapy201810.1177/2040206618770518
Data exclusions	No data was excluded
Replication	Each study is internally controlled with positive and negative controls.
Randomization	Animals were acquired and acclimated for at least one week upon arrival from the vendor at the West Point rodent vivarium. Animals were paired housed and assigned to a group randomly. Since there were an odd number of animals per group, there was always one cage per group that contained an animals from the adjacent group. Animals were identified via ear tag identification.
Blinding	The in-life part was not blinded as the dosing of each animals needed to be verified with the dosing vial assignment. Since the inoculation of the challenge virus was given equally to all groups (except for naïve control group), it was fitting to infect the animals in order. The harvesting of the respiratory tissue was done group order to avoid confusion, since each animals was treated equally there was no consideration to go out of order and since there was a cage per group containing animals from two independent groups, order was not considered for these cages as well. Ear tag identification was the only consideration, so each sample would be aligned with its corresponding, pre-labeled tube.

Reporting for specific materials, systems and methods

We require information from authors about some types of materials, experimental systems and methods used in many studies. Here, indicate whether each material, system or method listed is relevant to your study. If you are not sure if a list item applies to your research, read the appropriate section before selecting a response.

Materials & experimental systems

n/a	Involved in the study
☐	☒ Antibodies
☐	☒ Eukaryotic cell lines
☒	☐ Palaeontology
☐	☒ Animals and other organisms
☒	☐ Human research participants
☒	☐ Clinical data

Methods

n/a	Involved in the study
☒	☐ ChIP-seq
☒	☐ Flow cytometry
☒	☐ MRI-based neuroimaging

Antibodies

Antibodies used	CD3 BV421 PE Cy7 (cat. # 562426,BD Biosciences)- B cell sorting<br>anti-CD19 FITC (cat.# 555412,BD Biosciences)-B cell sorting<br>anti-IgG APC (Cat. # 550931, BD Biosciences)-B cell sorting

streptavidin-PE (cat# 349023BD Biosciences)-B cell sorting
RB1 was produced at GenScript according to the sequence, the CDR sequence is in the manuscript.
Horseradish peroxidase-conjugated goat anti-human IgG: source Southern Biotecc # 2040-05
Anti-RSV F murine antibody, clone 143-F3-B138, in house generated from our own hybridoma
Anti-RSV N murine antibody, clone 34C9, in house generated from our own hybridoma
Biotinylated horse anti-mouse IgG, vector laboratories, cat.#Cat# BA-2000
Palivizumab was procured at a medical supply vendor
Horseradish Peroxidase-Conjugated anti-Human IgG (for PK studies to detect RB1 in CR):Invitrogen 62-7120
RB1-LALA and Palivizumab-LALA were made at GenScript
anti-HMPV mab (EMD Millipore MAB80124) for HMPV neutralization assay
IgG Alexa 488 conjugated secondary antibody (Invitrogen #A11017) as a secondary in the HMPV neut. assay

Validation

RB1 lots produced at GenScript came with a certificate of analysis for sequence verification and were tested for binding to the RSV Fusion protein and RSV neutralization prior to use in experiments. In house anti-RSV F and anti-RSV N clones generated in house were tested for reactivity against the associated RSV protein in immunoassay prior to experiments with a irrelevant antibody as a negative control. RB1-LALA and palivizumab-LALA came with a certificate of analysis and QC from GenScript and were tested in an immunoassay and neutralization for binding to RSV F prior to the cotton rat study.

Eukaryotic cell lines

Policy information about cell lines

Cell line source(s)

HEp-2 (Human Epithelial Type 2), ATCC catalog number CCL-23. LLC-MK2, ATCC catalog number CCL-7.1

Authentication

The cell lines came from reputable vendors with certificate of analysis. No further authentication was performed. However, the behavior and morphology of the cells is routinely monitored.

Mycoplasma contamination

The cell lines were not tested for mycoplasma after we got them from the ATCC.

Commonly misidentified lines
(See ICLAC register)

HEp-2 is listed on the ICLAC list version 9. This cell line was used because RSV grows in these cells.

Animals and other organisms

Policy information about studies involving animals; ARRIVE guidelines recommended for reporting animal research

Laboratory animals

Cotton Rat, Sigmodon hispidus, female, 4-7 weeks,vendor: SAGE Labs Inc., Boyertown , Pennsylvania, USA or Envigo, Summerset NJ

Wild animals

None

Field-collected samples

None

Ethics oversight

Procedures involving the care and use of animals in the study were reviewed and approved by the Institutional Animal Care and Use Committee at Merck Research Laboratories. During the study, the care and use of animals were conducted in accordance with the principles outlined in the guidance of the Association for Assessment and Accreditation of Laboratory Animal Care (AAALAC), the Animal Welfare Act, the American Veterinary Medical Association (AVMA) Euthanasia Panel on Euthanasia, and the Institute for Laboratory Animal Research (ILAR) Guide to the Care and Use of Laboratory Animals.

Note that full information on the approval of the study protocol must also be provided in the manuscript.
